# A Self-Referenced Refractive Index Sensor Based on Gold Nanoislands

**DOI:** 10.3390/s23010066

**Published:** 2022-12-21

**Authors:** Carlos Angulo Barrios, Teona Mirea, Miguel Huerga Represa

**Affiliations:** 1 Department of Photonics and Bioengineering, CEMDATIC, ETSI Telecomunicación, Universidad Politécnica de Madrid, Ciudad Universitaria s/n, 28040 Madrid, Spain; 2 Department of Electronic Engineering, CEMDATIC, ETSI Telecomunicación, Universidad Politécnica de Madrid, Ciudad Universitaria s/n, 28040 Madrid, Spain

**Keywords:** optical sensor, refractive index, localized surface plasmon, metal nanoparticle

## Abstract

We report on a self-referenced refractive index optical sensor based on Au nanoislands. The device consists of a random distribution of Au nanoislands formed by dewetting on a planar SiO_2_/metal Fabry–Pérot cavity. Experimental and theoretical studies of the reflectance of this configuration reveal that its spectral response results from a combination of two resonances: a localized surface plasmon resonance (LSPR) associated to the Au nanoislands and the lowest-order anti-symmetric resonance of the Fabry–Pérot cavity. When the device is immersed in different fluids, the LSPR contribution provides high sensitivity to refractive index variations of the fluid, whereas those refractive index changes have little impact on the Fabry–Pérot resonance wavelength, allowing its use as a reference signal. The self-referenced sensor exhibits a spectral sensitivity of 212 nm/RIU (RIU: refractive index unit), which is larger than those of similar structures, and an intensity sensitivity of 4.9 RIU^−1^. The proposed chip-based architecture and the low cost and simplicity of the Au nanoisland synthesis procedure make the demonstrated sensor a promising self-referenced plasmonic sensor for compact biosensing optical platforms based on reflection mode operation.

## 1. Introduction

Refractive index (RI) sensors find important applications in disease diagnostics, biochemical analysis, environmental monitoring, and the agro-food industry. Among the different types of refractometric sensors, those based on surface plasmons (SPs) have attracted considerable attention as they offer remarkable features such as high sensitivity, easy miniaturization and parallelization, and integration feasibility [1,2,3,4,5,6]. SPs are formed by interactions between incident photons and free electrons on metal surfaces. Electromagnetic fields associated with SPs are strongly enhanced and confined to the metal surface, which makes the SP properties highly sensitive to variations of the RI of surrounding materials.

Most plasmonic RI sensors are based on the measurement of resonances related to either surface plasmon polaritons (SPPs) on thin metal films [1,3,7,8,9,10,11,12,13] or localized surface plasmons (LPS) on metal nanoparticles (NPs) [14,15,16,17,18]. SPP resonance measurements are traditionally carried out in the reflection mode using the Kretschmann or prism-based configuration [1,3,7,8,9], although other arrangements, such as optical waveguides and diffraction gratings, have been also reported [10,11,12,13]. SPP-based sensors have demonstrated good performance for refractometric monitoring; however, either large, complicated, and expensive optical equipment or highly specialized fabrication procedures are required. LSP-based sensors have also proved their suitability as RI sensors in numerous applications and can be integrated with microfluidic systems for lab-on-a-chip (LOC) devices. The optical intensity and spectral position of the LSP resonance band of a metal NP are characteristic of the type of material (typically, gold, silver, or platinum), size, distribution and shape of the nanostructure. Thus, the plasmonic properties of metal NPs can be tailored to meet the requirements of different applications, which is a major advantage of LSP-based sensors over SPP-based sensors. In addition, LSP sensors require simple and cost-effective readout equipment, such as a conventional spectrophotometer.

LSP-based RI sensing devices are usually implemented by using either colloidal solutions of metal NPs or chip-based designs in which nanostructures or nanoparticles are attached to a planar substrate. The chip-based approach has important advantages such as easier multiplexed analysis and integration with electronic and microfluidic systems. Chip-based LSP sensors can be fabricated by several means that can be classified into two general groups: lithography-based and lithography-free processes. Nanolithography techniques, such as electron beam lithography [19] and nanoimprint lithography [20], allow controlled positioning and dimensioning of nanostructures according to a specific pattern; however, they typically require multiple processing steps and complex, expensive processing facilities. On the other hand, lithography-free nanofabrication processes, such as colloidal nanoparticle deposition [21,22,23,24] and dewetting [25,26,27,28,29,30], typically result in a low-ordered or random distribution of nanoparticles, which exhibit significant size dispersion; nevertheless, they are simpler and lower-cost fabrication alternatives. In particular, thermal dewetting by annealing of thin metal films [25,26,27,28,29,30] is one of the easiest and cheapest methods to produce metal nanostructures on solid substrates. This process leads to the formation of metal nanostructures, usually called nanoislands, due to the dewetting or agglomeration of a thin metal layer occurring at an annealing temperature below the melting point of the metal while the film remains in a solid state [25]. Nanoislands dewetted from thin metal films can be massively formed at a wafer level and exhibit substrate-dependent plasmonic phenomena in a broad spectral range from ultraviolet to infrared [30].

Independently of the principle (SPP or LSP) on which a plasmonic RI sensor is based, external factors, such as vibrations and intensity fluctuations, may affect the sensing accuracy. To mitigate this issue, a variety of self-referenced RI plasmonic sensors have been proposed [31,32,33,34,35,36]. These are mostly based on the generation of independent multiple resonances with different sensitivities to the refractive index of the surrounding medium. In spectral interrogation schemes, self-referencing relies on finding the difference between the wavelengths of a resonance highly sensitive to the RI of the sample of interest and a reference resonance that is scarcely sensitive to changes of the sample RI. In intensity interrogation schemes, self-referencing is typically achieved by obtaining the quotient between the intensities of two resonances, both equally affected by light intensity fluctuations. Reported self-referenced plasmonic RI sensors include prism-based configurations [31,32] and grating-based structures [33,34,35,36]; however, to our knowledge, no self-referenced RI sensors based on metal nanoislands have been reported so far.

In this work, we propose and demonstrate a chip-based self-referenced refractive index plasmonic sensor based on Au nanoislands. The device consists of a random distribution of Au nanoislands formed on a semi-open SiO_2_/metal Fabry–Pérot cavity. Au nanoparticle LSP resonance provides spectral sensitivity to RI changes of the test sample, whereas the Fabry–Pérot structure leads to a resonance wavelength slightly affected by those RI variations, which makes it suitable as a reference. Au nanoislands have been fabricated using the low-cost and simple thermal annealing technique, and the device has been optically interrogated in reflection mode. A theoretical analysis based on finite difference time domain (FDTD) calculations has been also achieved in order to elucidate the physical origin of the measured spectral response.

## 2. Experimental Section

**Figure 1:**
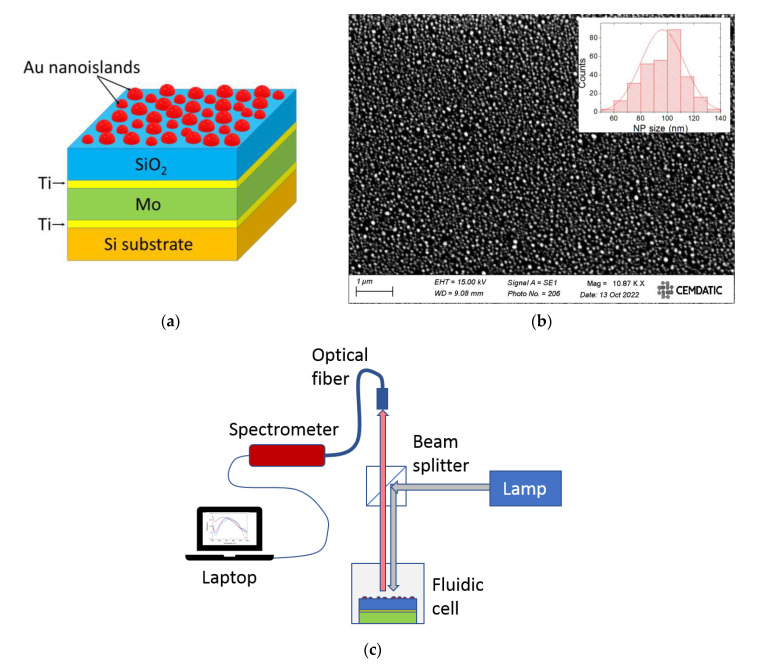
(**a**) Schematic view of the fabricated Au nanoisland/Fabry–Pérot cavity self-referenced refractive index sensor; (**b**) SEM top view of the fabricated device after thermal annealing revealing the formation of Au nanoislands (bright regions). Inset shows the size distribution histogram (bars) and normal function fit (line) of 300 Au NPs; (**c**) schematic diagram of the spectral reflectance measurement setup.

Au nanoislands were synthesized on a SiO_2_/Ti/Mo multilayer structure by thermal annealing. First, the multilayer was created on a Si substrate by subsequent deposition of a 10 nm-thick Ti layer, a 150 nm-thick Mo layer, a 10 nm-thick Ti layer and a 100 nm-thick SiO_2_ layer. All these layers were deposited in a DC-pulsed reactive sputtering system at 150 W with 5.4 mBar, 400 W with 0.7 mBar, and 1200 W with 3.3 mbar, for Ti, Mo, and SiO_2_, respectively. Next, a 15 nm-thick Au film was deposited on the SiO_2_ layer by Joule evaporation at a 5 Å/s rate. Finally, a thermal annealing process was carried out in a quartz oven for 2 h at 475 °C in an air atmosphere. Figure 1a,b show a schematic diagram and a top-view scanning electron microscope (SEM) photograph of the fabricated configuration, respectively. Figure 1b reveals that the formed Au nanoislands are randomly distributed and have an average size of approximately 100 nm (inset) and a surface density of approximately 60 nanoparticles/μm^2^.

The fabricated device was optically characterized in reflection mode, as schematically illustrated in Figure 1c. A collimated light beam from a tungsten-halogen lamp (Thorlabs SLS201L) was used to illuminate normally the surface containing the Au nanoislands by using a cube beam splitter. Light reflected from the device was collected by an optical fiber and spectrally analyzed by a monochromator (Thorlabs CCS200). Reflection from an Al mirror was used as a reference for determining the relative specular reflectance of the device. Refractive index sensing characterization was carried out at room temperature by immersing the device in air (RI = 1), distilled water (RI = 1.333), ethanol (RI = 1.362), isopropanol (RI = 1.375), and glycerol (RI = 1.465) inside a fluidic cell. The refractive indexes of the liquids were measured with an Abbe refractometer. The sensor response repeatability was determined by acquiring five consecutive measurements for each refractive index. The sensor response reproducibility was addressed by obtaining 20 pairs of measurements, each pair consisting of measuring first in air, and next in distilled water. After each air-water measurement sequence, the chip sensor was removed from the characterization setup, cleaned with compressed air, and placed again on the setup holder for a subsequent measurement pair.

## 3. Optical Simulations

**Figure 2:**
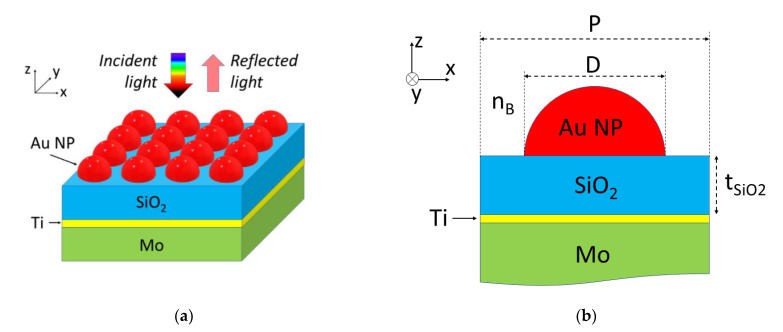
(**a**) Schematics of the modeled self-referenced plasmonic sensor based on Au nanoislands. The sensor is interrogated in reflection mode; (**b**) schematic cross-sectional view of the simulated device. The thickness of the SiO_2_ layer, t_SiO2_, equals 100 nm.

Figure 2 shows a schematic diagram of the simulated configuration. Hemispherical Au nanoparticles of diameter D are arranged in a square array of period P. The Au nanoparticles rest on a 100 nm-thick SiO_2_ layer, which lies on a 10 nm-thick Ti layer deposited on a semi-infinite Mo substrate. The structure is surrounded by a bulk material (test sample) of refractive index n_B_. The frequency dependent dielectric constant of Au, Ti, and Mo was modeled by the well-known Drude−Lorentz equation with the fitted parameters reported in the literature [37,38]. The dielectric constant of SiO_2_ was assumed to be frequency-independent and equal to 1.455. The reflection spectrum and field calculations of the modeled device were calculated by the FDTD algorithm [39]. Periodic boundary conditions were chosen along the device plane coordinates (*x*- and *y*-axis) and perfectly matched layer (PML) boundary condition was used along the incident-beam propagation direction (*z*-axis), normal to the device plane. The grid of the simulation domain was non-uniform, with grid size = 5 nm in the bulk regions and grid size = 1 nm near material interfaces in the x-, y-, and *z*-axis. Frequency analysis of the reflection was achieved by launching a pulsed excitation from the top region towards the Au nanoislands and calculating the fast Fourier transform (FFT) of the reflected time-domain field component (E_x_) on a plane above the nanoislands.

## 4. Results

**Figure 3:**
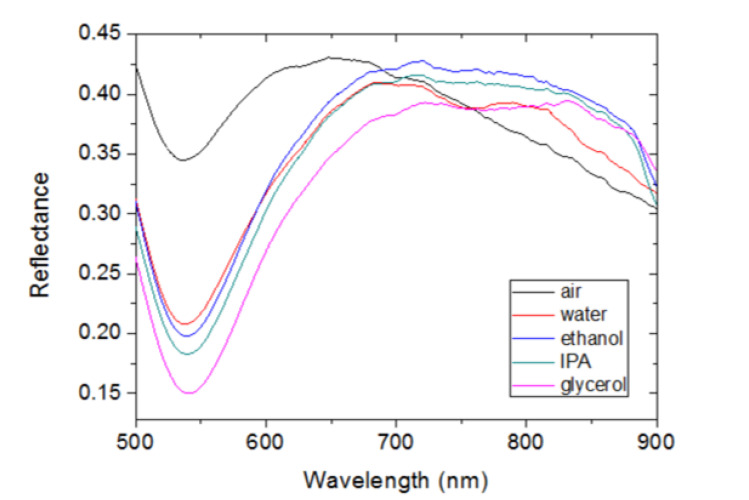
Spectral reflectance of the fabricated device when exposed to different fluids: air (black line), water (red line), ethanol (blue line), isopropanol (green line), and glycerol (magenta line).

**Figure 4:**
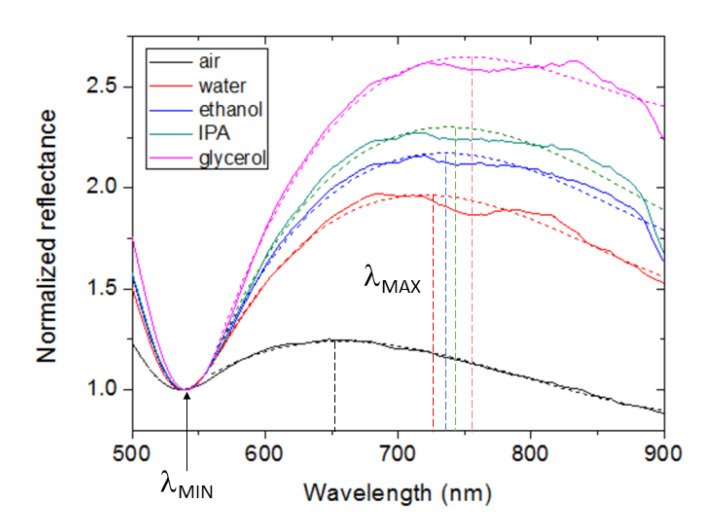
Spectral reflectance curves for different fluids normalized to the reflectance minimum (solid lines) and corresponding third degree polynomial fittings in the 560–900 nm wavelength range (dotted lines). Vertical dashed lines indicate the spectral position of the maximum reflectance (λ_MAX_) of the fittings for each fluid. λ_MIN_ denotes the reflectance dip wavelength.

Figure 3 shows the measured spectral reflectance (R) of the fabricated device in the 500–900 nm spectral range when exposed to air (black line), water (red line), ethanol (blue line), isopropanol (green line), and glycerol (magenta line). It can be observed that all curves exhibit a dip at approximately 540 nm wavelength and a broad band or “hill” at longer wavelengths. Note that the dip wavelength (λ_MIN_) is scarcely affected by the fluid refractive index, whereas the spectral hills appear to shift to the red as the refractive index of the fluid increases. Spectral shifts are typically recorded by monitoring the wavelength corresponding to a maximum or a minimum of the device response. In the measured curves of Figure 3, no clear maximum value is observed, particularly when the device is exposed to liquids, due to the non-smooth variation of the reflectance at the top of the hills. To circumvent this issue, the experimental curves were fitted by third degree polynomial functions in the 560–900 nm spectral range, and the wavelengths corresponding to the maximum reflectance values of the fittings (λ_MAX_) were used to quantify the redshift. Figure 4 shows the experimental curves normalized to R(λ_MIN_) and the corresponding polynomial fittings. Figure 4 depicts both the little impact of the fluid refractive index on the dip spectral position and the redshift of the spectral bands.

**Figure 5:**
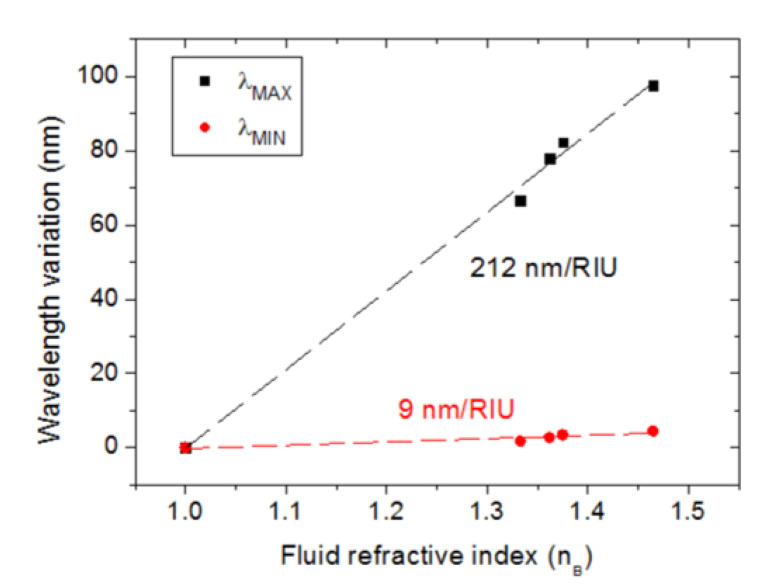
Variation of λ_MAX_ and λ_MIN_ as a function of the fluid refractive index (n_B_). Dashed lines represent linear fits of the measured data points. Error bars are smaller than dots.

Figure 5 shows the variations of λ_MAX_ and λ_MIN_ with the fluid refractive index. Linear fittings of the data led to sensitivity values of 212 nm/RIU and 9 nm/RIU for λ_MAX_ and λ_MIN_, respectively. That is, the sensitivity of λ_MAX_ to the bulk refractive index is 23 times greater than that of λ_MIN_. This large sensitivity ratio allows the use of λ_MAX_ and λ_MIN_ as the sensing and reference signals, respectively. For the sake of comparison, Sun et al. [35] proposed and modeled a self-referenced RI sensor based on a dielectric-metal 2D grating structure displaying a sensing-to-reference sensitivity ratio of 15, which is smaller than that of our device. The RI resolution or limit of detection (LOD) can be defined as LOD = 3σ/S, where S is the sensitivity and σ is the standard deviation for the blank sample (distilled water for aqueous solutions). From five consecutive measurements, σ turned out to be equal to 0.2 nm, which indicates an LOD of 3 × 10^−3^ RIU at the refractive index of water. The reproducibility study revealed measurement uncertainties (standard deviations) of 0.6 nm and 5.4 nm for λ_MIN_ and λ_MAX_, respectively, in the case of air, and 0.5 nm and 4.2 nm, respectively, in the case of distilled water.

**Figure 6:**
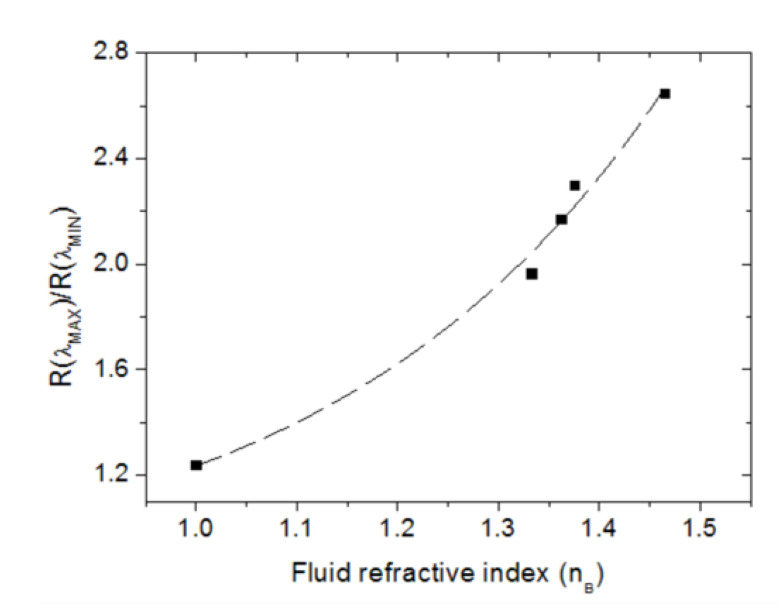
Normalized reflectance R(λ_MAX_)/R(λ_MIN_) as a function of the fluid refractive index (n_B_). Dashed line represents an exponential fit of the measured data points. Error bars are smaller than dots.

Figure 4 also shows a clear increment of the magnitude of the normalized band reflectance with the fluid refractive index. This suggests an alternative method to monitor the bulk refractive index. In this case, the analytical signal could be defined as R(λ_S_)/R(λ_MIN_), where λ_S_ is a wavelength within the spectral hill at which the reflectance varies significantly with n_B_; for example, λ_S_ = λ_MAX_. Note that the effect of intensity variations of the light source can be highly reduced by using this relative measurement, since both R(λ_S_) and R(λ_MIN_) would be equally affected. Figure 6 plots R(λ_MAX_)/R(λ_MIN_) as a function of the fluid refractive index. The experimental data points have been fitted by a single exponential function (dashed line). Assuming a linear variation around the refractive index of water, the RI sensitivity equals 4.9 RIU^−1^. From five consecutive measurements, σ = 0.003, which leads to an LOD of 2.4 × 10^−3^ RIU, which is similar to that obtained by monitoring spectral shifts.

**Figure 7:**
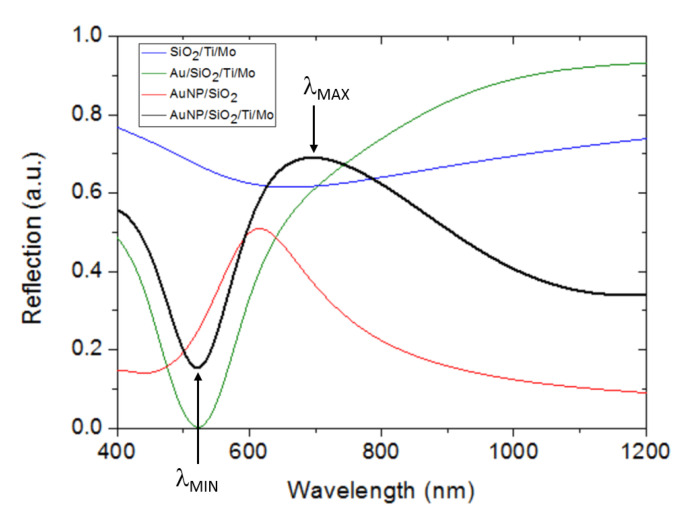
Calculated spectral reflection for different multilayer configurations: SiO_2_ (100 nm)/Ti (10 nm)/Mo substrate (blue line), Au (15 nm)/SiO_2_ (100 nm)/Ti (10 nm)/Mo substrate (green line), Au NP (100 nm diameter)/SiO_2_ substrate (red line), and Au NP (100 nm diameter)/SiO_2_ (100 nm)/Ti (10 nm)/Mo substrate (black line). In all cases n_B_ = 1 (air).

FDTD calculations were carried out in order to gain insight into the nature of the measured spectral reflectance. The FDTD-calculated spectral reflection of the modeled configuration (Figure 2) for D = 100 nm, P = 130 nm, and n_B_ = 1 is shown in Figure 7 (black line). As with the corresponding experimental curve, two relevant spectral features are observed: a dip with its minimum at λ_MIN_ = 521.5 nm and a broad band or hill with its maximum at λ_MAX_ = 697 nm. The spectral reflections of three related planar structures were also calculated and plotted in Figure 7: a 100 nm-thick SiO_2_ layer on Ti (10 nm)/Mo substrate (blue line), a 15 nm-thick Au film on SiO_2_ (100 nm)/Ti (10 nm)/Mo substrate (green line), and a square lattice (period = 130 nm) of 100 nm diameter hemispherical Au NPs on a SiO_2_ substrate (red line). It can be observed that both the SiO_2_/Ti/Mo and Au/SiO_2_/Ti/Mo structures show a reflection minimum at 663.6 nm and 524.3 nm, respectively. These dips can be attributed to the lowest order Fabry–Pérot resonance of the cavity formed by the SiO_2_ layer and the high reflective metal substrate (Ti/Mo). Note that the presence of a thin film of Au on the SiO_2_ layer increases the reflectance of the top surface of the Fabry–Pérot cavity, leading to a deeper and narrower resonance reflection dip. On the other hand, the Au NP/SiO_2_ structure exhibits a reflection peak at 699 nm wavelength, which can be attributed to an LSP resonance of the Au NPs [40]. These responses indicate that the Au NP/SiO_2_/Ti/Mo configuration supports an LSP resonance associated with the Au NPs in the vicinity of the lowest order resonance of the SiO_2_/metal Fabry–Pérot nanocavity. Thus, its overall response could be understood as the interaction or hybridization between those two basic resonance modes [41].

**Figure 8:**
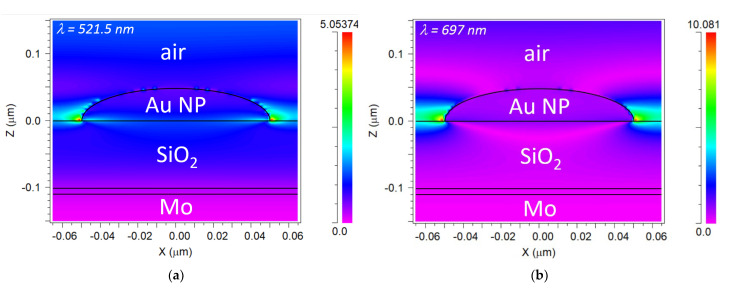

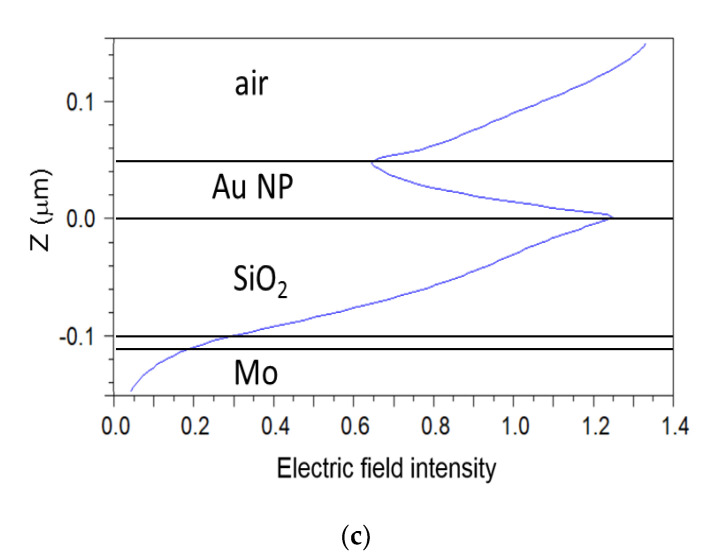
Calculated electric field (E_x_) distribution at λ_MIN_ = 521.5 nm (**a**) and λ_MAX_ = 697 nm (**b**) of the modeled Au NP/SiO_2_/Ti/Mo configuration in the XZ plane; (**c**) x-cut along x = 0 of (**a**).

To corroborate the previous statements concerning the origin of the spectral features of interest, the electric field (E_x_) distributions of the Au NP/SiO_2_/Ti/Mo configuration at λ_MIN_ = 521.5 nm and λ_MAX_ = 697 nm were calculated and shown in Figure 8a,b, respectively. Figure 8c shows an x-cut (x = 0) of the 2D field distribution of Figure 8a. It is shown in Figure 8c that the field intensity profile at λ_MIN_ = 521.5 nm decays monotonically in the SiO_2_ layer, which is characteristic of an anti-symmetric Fabry–Pérot resonance [42]. The absence of nodes indicates that only the lowest order Fabry–Pérot mode is involved. On the other hand, Figure 8b shows that the E_x_ field intensity at λ_MAX_ = 697 nm is highly enhanced at the right and left edges of the Au NP, which is a typical behavior of an LSP resonance.

**Figure 9:**
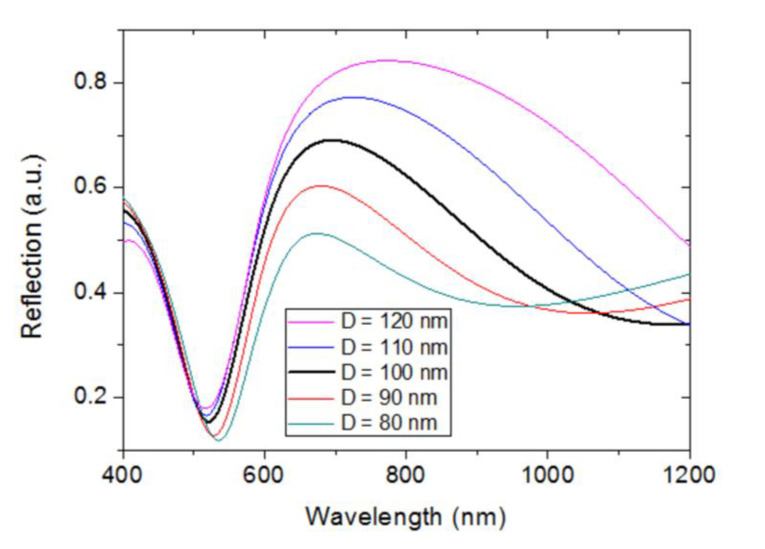
Calculated spectral reflections of the modeled Au NP/SiO_2_/Ti/Mo configuration for different values of the Au NP diameter (D). The array period (P) equals 130 nm.

Further corroboration is provided by Figure 9, which plots the calculated spectral reflection of the proposed configuration for different Au NP diameters (D), ranging from 80 to 120 nm. In all cases the period of the NP array equals 130 nm. Figure 9 shows that the NP diameter affects both λ_MAX_ and λ_MIN_. λ_MAX_ increases as D increases, as expected from an LSP resonance associated to a metal NP [28]. On the other hand, λ_MIN_ decreases as D increases, in agreement with the performance of an Au/SiO_2_/Ti/Mo Fabry–Pérot cavity in which the reflectivity of the Au/SiO_2_ interface increases due to the increment of the filling factor of the Au NP array. The effect of D variation on λ_MAX_ is more significant than that on λ_MIN_; this is because the latter is also highly dependent on the thickness of the SiO_2_ layer, which is constant. This may explain the greater measurement uncertainty of λ_MAX_ (~4–5 nm), compared with that of λ_MIN_ (~0.5–0.6 nm), obtained in the reproducibility study of the actual device. In those experiments, described in Section 2, repositioning of the sensor chip between measurements might lead to small deviations of the sensor position in the XY plane from one measurement to another. This would modify the Au NP size distribution illuminated by the interrogation beam and, therefore, introduce greater uncertainty in the measure of λ_MAX_, which is more sensitive to the NP size than λ_MIN_.

**Figure 10:**
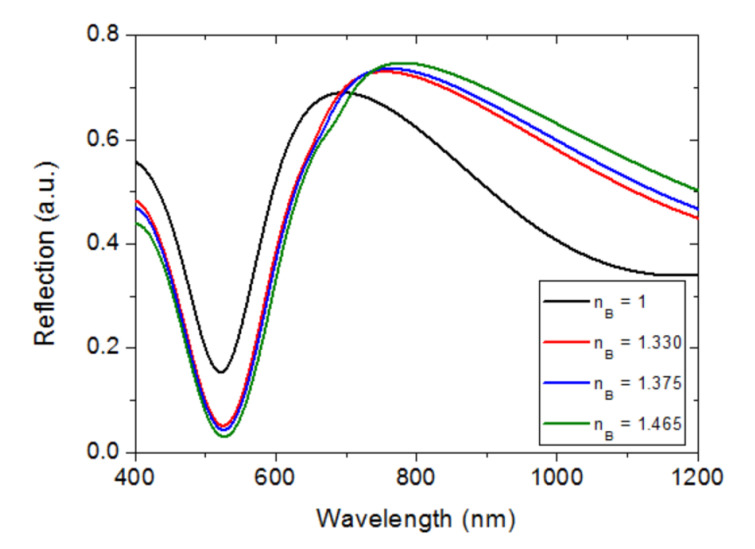
Calculated spectral reflections of the modeled Au NP/SiO_2_/Ti/Mo configuration for different values of the bulk refractive index n_B_.

**Figure 11:**
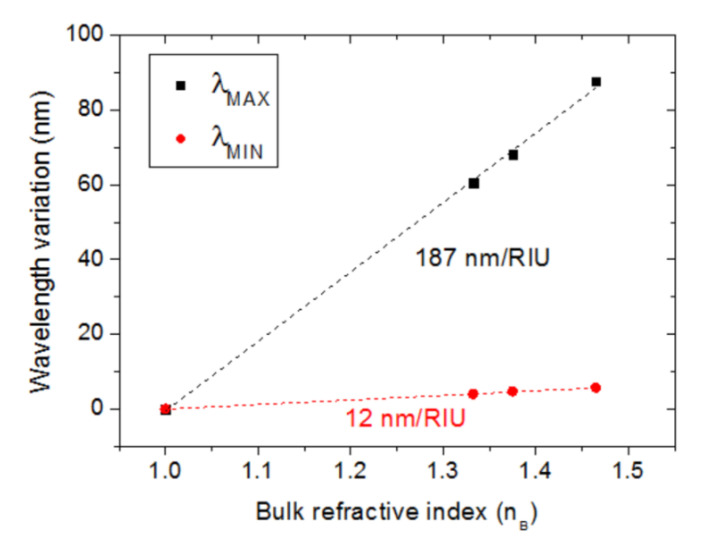
Calculated λ_MAX_ and λ_MIN_ variations as a function of the bulk refractive index (n_B_).

Figure 10 shows the calculated spectral reflection of the modeled configuration for different values of the bulk refractive index. It is seen that the shapes of the calculated curves resemble those obtained experimentally. The dip spectral position is not greatly affected by n_B_ variations, as expected, since the electric field at the Fabry–Pérot resonance wavelength is mainly confined in the SiO_2_ layer. On the other hand, the reflection maximum, related to the LSP resonance of the Au NPs, displays a significant variation with n_B_ because of the interaction of the electric field with the surrounding bulk material. Figure 11 shows the variations of the calculated λ_MAX_ and λ_MIN_ as a function of n_B_. The resulting bulk refractive index sensitivities were 187 nm/RIU and 12 nm/RIU for λ_MAX_ and λ_MIN_, respectively, which compare well with the corresponding experimental values.

## 5. Discussion

RI sensitivities exhibited by reported LSP sensors based on Au NPs deposited on non-layered conventional supports, such as Si, SiO_2_, and Si_3_N_4_, are around 20–60 nm/RIU [24,26,29]. As demonstrated by Ferhan et al. [43] and in this work, greater sensitivities can be achieved by using a highly reflective substrate and conducting LSP resonance measurements in reflection mode. In particular, the RI sensitivity reported in [43] was 150 nm/RIU. The sensor demonstrated here shows higher sensitivity and offers self-referencing functionality. Although the monitored RI-sensitive spectral band is wide and non-smooth, a simple polynomial fit of the measured curve provides a reliable method to quantify the spectral shifts. In addition, reflection mode interrogation allows the integration of plasmonic Au nanoislands on structures containing opaque metal electrodes. For example, Au nanoislands could be synthesized on the top electrode of a conventional quartz crystal microbalance (QCM) sensor with minimal modification for simultaneous electro-acoustic and optical biosensing [43].

It should be also noted that the simulated device consists of an ordered array of Au hemispheres of fixed diameter, whereas the experimental device is formed by randomly distributed Au nanoislands of non-uniform size and shape. This may explain the non-smooth and wider bands exhibited by the experimental configuration (Figure 3 and Figure 4) as compared to those of the modeled device (Figure 10). In particular, the numerous nanogaps of different lengths formed by adjacent metal nanoislands in the fabricated structure can lead to interparticle coupling effects that can be manifested as a broader and non-smooth spectral band [28,30]. Nevertheless, despite these morphological differences, both structures exhibit similar spectral responses and comparable RI sensitivities for both the reference and the sensing signals. This makes the proposed theoretical model suitable for design optimization of Au nanoisland-based sensor architectures. Furthermore, it suggests that a simple and cost-effective fabrication method such as thermal annealing can produce devices with similar performance as those manufactured by using expensive and complex nanolithography techniques.

## 6. Conclusions

A self-referenced refractive index sensor based on Au nanoislands deposited on a semi-open SiO_2_/metal Fabry–Pérot nanocavity has been proposed and demonstrated. Au nanoislands have been synthesized on a SiO_2_/metal support by thermal dewetting. Both experimental measurements and computer simulations reveal that the reflectance of the device results from the combination of two resonance phenomena: a Fabry–Pérot resonance associated with the SiO_2_/metal nanocavity and an LSP resonance related to the Au nanoislands. The former/latter is minimally/highly sensitive to bulk refractive index variations and can therefore be used as a reference/sensing signal. The sensitivity of the fabricated device is 212 nm/RIU, which is higher than that of similar configurations based on Au nanoparticles deposited on planar substrates, while offering the additional advantage of self-referencing. These performance improvements and the low cost and simplicity of the Au nanoislands synthesis procedure make the demonstrated device a promising plasmonic sensor for integration into chip-based biosensing optical platforms, particularly in those containing thick metal layers that require reflection mode operation.

## Data Availability

Data are contained within the article.
